# Bone remodeling and responsiveness to mechanical stimuli in individuals with type 1 diabetes mellitus

**DOI:** 10.1093/jbmr/zjad014

**Published:** 2024-01-04

**Authors:** Matthias Walle, Ankita Duseja, Danielle E Whittier, Tatiane Vilaca, Margaret Paggiosi, Richard Eastell, Ralph Müller, Caitlyn J Collins

**Affiliations:** Institute for Biomechanics, ETH Zurich, Zurich, Switzerland; Department of Oncology and Metabolism, University of Sheffield, Sheffield, United Kingdom; Institute for Biomechanics, ETH Zurich, Zurich, Switzerland; Department of Osteoporosis, Bern University Hospital, University of Bern, Bern, Switzerland; Department of Oncology and Metabolism, University of Sheffield, Sheffield, United Kingdom; Department of Oncology and Metabolism, University of Sheffield, Sheffield, United Kingdom; Department of Oncology and Metabolism, University of Sheffield, Sheffield, United Kingdom; Institute for Biomechanics, ETH Zurich, Zurich, Switzerland; Institute for Biomechanics, ETH Zurich, Zurich, Switzerland; Department of Biomedical Engineering and Mechanics, Virginia Tech, Blacksburg, VA, United States

**Keywords:** type 1 diabetes mellitus, neuropathy, high–resolution peripheral quantitative–computed tomography, micro–finite element analysis, mechanoregulation, bone remodeling

## Abstract

Type 1 diabetes mellitus (T1DM) has been linked to increased osteocyte apoptosis, local accumulation of mineralized lacunar spaces, and microdamage suggesting an impairment of the mechanoregulation network in affected individuals. Diabetic neuropathy might exacerbate this dysfunction through direct effects on bone turnover, and indirect effects on balance, muscle strength, and gait. However, the *in vivo* effects of impaired bone mechanoregulation on bone remodeling in humans remain underexplored. This longitudinal cohort study assessed consenting participants with T1DM and varying degree of distal symmetric sensorimotor polyneuropathy (T1DM, *n* = 20, median age 46.5 yr, eight female) and controls (CTRL; *n* = 9, median age 59.0 yr, four female) at baseline and 4–yr follow-up. Nerve conduction in participants with T1DM was tested using DPNCheck and bone remodeling was quantified with longitudinal high–resolution peripheral quantitative–computed tomography (HR-pQCT, 82 μm) at the standard distal sites. Local trabecular bone formation (Tb.F) and resorption (Tb.R) sites were captured by implementing 3D rigid image registration of HR-pQCT images, and the mechanical environment across the bone microarchitecture at these sites was simulated using micro–finite element analysis. We calculated odds ratios to determine the likelihood of bone formation (OR_F_) and resorption (OR_R_) with increasing/decreasing strain in percent as markers for mechanoregulation. At the distal radius, Tb.F was 47% lower and Tb.R was 59% lower in T1DM participants compared with CTRL (*P* < .05). Tb.F correlated positively with nerve conduction amplitude (*R* = 0.69, *P* < .05) in participants with T1DM and negatively with glycated hemoglobin (HbA1c) (*R* = −0.45, *P* < .05). Additionally, OR_F_ was 34% lower and OR_R_ was 18% lower in T1DM compared with CTRL (*P* < .05). Our findings represent *in vivo* evidence suggesting that bone remodeling in individuals with T1DM is in a state of low responsiveness to mechanical stimuli, resulting in impaired bone formation and resorption rates; these correlate to the degree of neuropathy and level of diabetes control.

## Lay Summary

In a healthy adult, the body’s skeleton self-repairs—or remodels—itself to maintain its strength. At the microscopic level, this process is orchestrated by cells, called osteocytes, which can sense and respond to local mechanical forces. Recent studies have suggested that type 1 diabetes mellitus (T1DM), a metabolic bone disease, may negatively impact this mechanically regulated process and reduce bone strength. To investigate this further, we utilized novel methods to monitor local changes in bone microstructure over time using high–resolution peripheral quantitative–computed tomography, allowing us to study the results of cellular behavior on bone remodeling in participants over time. Our study found that bone formation was 47% lower and bone resorption was 59% lower in participants with T1DM compared with controls (CTRL). Bone formation correlated positively with peripheral nerve function and negatively with glycaemic control in participants with T1DM. Furthermore, the links between mechanical forces acting on bone remodeling were 34% weaker for formation and 18% weaker for resorption compared with CTRL. Our findings show that bone remodeling in people with T1DM is in a state of low responsiveness to mechanical stimuli, resulting in impaired bone formation and resorption rates, and ultimately, impaired self-repair.

## Introduction

Current understanding of increased bone fragility in patients with type 1 diabetes mellitus (T1DM) suggests that a decrease in the number, function, and maturity of bone–forming cells known as osteoblasts is the primary driver.^1–3^ However, recent advances in the field of bone biology have highlighted the critical role of osteocytes in maintaining bone homeostasis, emphasizing the importance of these cells as contributors to bone T1DM pathophysiology.^4^^,^^5^ Given that mechanical loading is an essential anabolic stimulus for bone health maintenance, impaired osteocyte mechanoregulation and signal transduction have been proposed as central to the mechanisms that lead to low bone turnover and bone loss in T1DM.^6^^,^^7^ Emerging research suggests that hyperglycaemia may exert deleterious effects on mechanosensitive membrane channels, thereby causing a substantial downregulation of mechano–signaling proteins, as evidenced in an in vivo murine model of T1DM and *in vitro* experiments exposing osteocytes to high glucose levels.^6^^,^^8^ This resultant downregulation impairs the ability of mechanically induced adenosine triphosphate signaling and regulation of osteocyte apoptosis, thereby implying that bones of individuals with T1DM may respond differently to mechanical stimuli such as physical or weight–bearing activity. Moreover, prevalent complications of T1DM, such as distal symmetrical sensorimotor polyneuropathy (DSPN), are frequently linked to persistent hyperglycaemia and adverse effects on balance, muscle strength, and gait.^9^ In the context of DSPN, the activation of tropomyosin receptor kinase A receptors in sensory nerves within bone tissue may be adversely affected, leading to the modulation of osteoblast activity, decreased production of bone–building factors, and attenuation of the Wnt/β–catenin signaling pathway, which are vital for bone formation.^10^^,^^11^ Ultimately, impaired bone mechanoregulation may lead to the deposition of bone in regions that provide less mechanical benefit, while simultaneously removing bone in mechanically more fragile areas. We hypothesized the combined effect of impaired mechanoregulation and decreased bone turnover may be the underlying cause of the skeletal dysfunction and altered bone microstructure frequently observed in individuals with T1DM, especially those with DSPN.

Although mechanoregulation has not been investigated in patient cohorts with T1DM, our recent meta-analysis revealed more pronounced alterations in bone microarchitecture among individuals with T1DM at the non–weight–bearing radius compared with the weight–bearing tibia.^12^ From these findings, we hypothesized that consistent mechanical stimulation might be crucial for preserving regular bone microarchitecture in individuals with T1DM. To directly evaluate bone mechanoregulation *in vivo*, we have developed a computational method using longitudinal high–resolution peripheral quantitative–computed tomography (HR-pQCT) based on prior evidence for load–driven remodeling in humans.^13^^,^^14^ We demonstrated that this technique could identify remodeling sites with exceptional precision, allowing for the detection of subtle changes that can be linked to bone cell activity in humans.^15^ In addition, we developed a computational load estimation algorithm using longitudinal HR-pQCT images to simulate the local mechanical environment under habitual loading conditions.^16^ Combined, these approaches enable observed bone remodeling sites *in vivo* to be correlated with physiological mechanical stimuli, and thus the degree of mechanically driven bone remodeling can be quantified.

The aim of this study was to investigate the effect of T1DM and DSPN on bone mechanoregulation. In the context of this work, we investigate local mechanoregulation that drives microstructural bone adaptation by resorbing bone below and forming bone above certain tissue–level strain thresholds, to increase load–bearing strength.^17^^,^^18^ To achieve this aim, we identified bone formation and resorption sites in participants with T1DM and varying degree of DSPN, alongside healthy age– and sex–matched controls (CTRL), using longitudinal HR-pQCT imaging at the distal radius and tibia. We quantified the local mechanical stimuli at bone remodeling sites by utilizing a load estimation algorithm through participant–specific micro–finite element (micro-FE) analysis. Furthermore, we investigated the correlation between mechanical stimuli and bone remodeling sites in these groups. We generated odds ratios for formation (OR_F_) and resorption (OR_R_) and a correct classification rate (CCR) of bone mechanoregulation, which could be used to assess whether mechanoregulation was impaired in individuals with T1DM. Specifically, odds ratios quantify the spatial correlation between local bone formation events and local strain levels. Low OR_F_ would indicate bone formation in mechanically less significant (low strain) locations, whereas low OR_R_ would imply the resorption of bone in more fragile (high strain) areas. These findings have the potential to provide initial mechanistic evidence translating previous cellular evidence for impaired mechanoregulation to an *in vivo *population and may have implications for understanding bone fragility in T1DM.

## Materials and methods

### Participants and image acquisition

This study conducted a longitudinal follow-up of the cohort from a previous single–center, observational, case–controlled study^9^ aimed at evaluating the effects of T1DM and diabetic neuropathy on the skeleton in participants with T1DM. We previously acquired HR-pQCT and clinical data from participants who met the following inclusion criteria: Caucasian adults with T1DM and estimated glomerular filtration rate > 60 ml/min/1.73 m^2^ and healthy CTRL over 18 yr of age. Participants were recruited from diabetes clinics and research participant lists in Sheffield, UK, between October 2017 and 2018 and re-recruited between August 2022 and December 2022. For this present study, only 32 out of the 60 participants could be re-recruited for follow–up visits because of the inability to attend appointments. One participant was excluded from the study because of their use of antiresorptive drugs and two were excluded because of menopause (<5 yr). Therefore, the present study sample consisted of 20 individuals with T1DM (with and without neuropathy), and nine skeletally healthy CTRL. All participants underwent thorough clinical and neurophysiological assessments,^19^ as previously described.^9^ The degree of neuropathy was identified using the Toronto Clinical Neuropathy Score and nerve conduction assessment by DPNCheck (Neurometrix, Waltham, MA, USA). We assessed fasting biochemical bone turnover markers (N-terminal propeptide of type I collagen (PINP), C-terminal telopeptide of type I collagen (CTX-I)) in plasma in a single batch using the IDS-iSYS multidisciplined automated chemiluminescence immunoassay (Immunodiagnostic Systems, Boldon, UK). The interassay CVs were 5.4% for CTX and 9.1% for PINP. To analyze handgrip strength, a digital hand dynamometer (Seahan Corp., Masan, South Korea) was employed, and three measurements were taken on each side. The maximum overall grip strength recorded was utilized for further analysis. This study was approved by the London-Harrow Research Ethics Committee (IRAS 303770, 21/PR/1712), and all participants provided written informed consent in accordance with Good Clinical Practice guidelines.

We used HR-pQCT (XtremeCT, Scanco Medical AG, 82 μm) at the nondominant radius and tibia at baseline and ~4–yr follow-up in all participants. HR-pQCT scans were acquired following the manufacturer’s standard in vivo protocol.^20^ In brief, a reference line was placed on the distal radial or tibial joint surface using anteroposterior scout views. The scan region (110 slices) was offset 9.5 and 22.5 mm from a reference line placed at the inflection point on the endplate of the distal radius or tibial plafond, respectively. The periosteal contour was automatically identified using 3D geodesic active contours for automatic segmentation.^21^ The endocortical contour was automatically identified in all images using the dual–threshold technique (cortical bone: 450 mg HA/cm^3^, trabecular bone: 320 mg HA/cm^3^).^22^ Contours were visually inspected for notable deviations from the periosteal or endocortical surfaces and manually corrected where necessary.^23^ Scans were automatically graded for motion artifacts using a previously developed motion–scoring algorithm on a 5–point scale (1 = no motion to 5 = large discontinuities in cortical structure),^24^ with manual verification. Participants were included in the study if they had attended both visits and all scans had a motion score of 3 or lower.

### Bone formation and resorption fractions

Bone formation and resorption sites were identified using a previously developed approach.^15^ In brief, the baseline and follow–up images were aligned by optimizing Euler angles and maximizing the voxel–wise correlation between grayscale density values within the periosteal contour. A Powell optimization algorithm was used with a five–level pyramid registration framework. Grayscale images were transformed using linear interpolation, and a Gaussian filter was applied to reduce noise. Binary segmentations of the bone were generated using adaptive local thresholding^25^ and transformed using nearest–neighbor interpolation. The common trabecular region across baseline and follow–up scans was determined from the registered images to exclude voxels outside the common region. Segmented images were then superimposed to identify regions of formation and resorption in the trabecular compartment. To reduce the detection of false remodeling events, the identified formation and resorption sites were further filtered, requiring a minimum density change of 225 mg HA/cm^3^ and a minimum cluster size of five voxels.^26^ Trabecular bone formation (Tb.F) and resorption (Tb.R) volumes were expressed as percent fraction of the baseline trabecular bone volume. Additionally, a net remodeling rate (Tb.F–Tb.R) was determined by subtracting the percent volume of Tb.R from the percent volume of Tb.F.

### Bone mechanoregulation

Bone mechanoregulation was assessed using a previously developed method.^15^ In brief, a physiological load estimation algorithm was used to estimate the local mechanical signal in the bone tissue of the baseline scan.^16^ The algorithm estimates physiological loading by superimposing three independent finite element (FE) calculations.^27^ This involved generating FE meshes by converting all voxels to eight–node hexahedral elements and assigning a Young’s modulus of 8.748 GPa and Poisson’s ratio of 0.3.^28^ Three orthogonal load cases of up to 1% apparent strain were then calculated, and linear micro-FE calculations were solved using ParOsol at the ETH research computing cluster (Euler). Using a Nelder–Mead method, strain energy density (SED) was maximized where bone was formed and minimized in regions of resorption. To link this mechanical signal to the remodeling events detected on the bone surface between baseline and the 1–yr follow-up, participant–wise conditional probability curves were computed. These curves analyzed the probability of bone remodeling events at various strain levels, with SED normalized using the 99th percentile and binned at 1% intervals. The conditional probability curves were then used to calculate the CCR, which estimates the proportion of remodeling events (resorption, quiescence, and formation) correctly classified relative to the mechanical signal. However, to independently assess the extent to which formation and resorption events are load-driven, logistic regression was performed. This was done to ascertain the participant–specific association between the mechanical signal (baseline SED) and voxel–wise bone formation and resorption. The resulting odds ratios for bone resorption (OR_R_) and formation (OR_F_), with 99% confidence intervals (CI), were computed per 1–percentage–point change in normalized mechanical signal (SED/SED_max_) to quantify strain–driven bone formation and resorption in individual participants. A CI of 99% was used because of the large sample size when performing voxel–wise analysis.

### Statistical analysis

Bone mechanoregulation, quantified by OR_R_ and OR_F_, was the main outcome of interest in this study. Based on our previous precision study reporting OR_R_ of 2.0 (99% CI: 1.8-2.2) and OR_F_ of 1.9 (99% CI: 1.7-2.1)^15^ in a healthy population, we estimated in this exploratory study that a sample size of seven per group has 80% power to detect a difference of 25% in OR_R_ and OR_F_ at *P* < .05.

Normally distributed variables were reported as mean and standard deviation, whereas non–normally distributed variables were presented as median and interquartile range. Student’s *t*-test was used to compare normally distributed variables, whereas the Wilcoxon–Mann–Whitney test was used to compare non–normally distributed variables. The correlation between HbA1c and bone remodeling fractions and mechanoregulation was assessed using linear regression analysis. A *P*-value < .05 was considered statistically significant for these analyses, with two–tailed testing. All statistical analyses were performed using Python (v3.8.5).

## Results

The present study sample consisted of 20 T1DM, and nine CTRL individuals with a mean age of 49.2 (±12.7) yr, including 41% females. The average weight and height of the participants were 72.4 (±14.5) kg and 170.1 (±10.4) cm, respectively. Furthermore, five tibia and eight radius scans were excluded because of participant motion during scanning at baseline or follow-up.

Study population characteristics for individuals only assessed at the radius or tibia are reported in Supplementary Tables S1 and S2, respectively. There were no significant differences between the groups of participants in terms of age, sex, weight, and height, with *P*-values of .13, 1.00, .48, and .42, respectively (Table 1**).** These results indicate that the study population was well-balanced in terms of demographic factors and that any observed differences between groups are likely because of disease–related factors rather than demographic differences between groups.

**Table 1:** Study population characteristics, biochemical bone turnover markers, and neuropathy assessment as mean (standard deviation) at baseline for normally distributed variables and median (interquartile range) for non–normally distributed variables.

	T1DM (*n* = 20)	CTRL (*n* = 9)	*P*-value
Age [yr]	46.5 (36.0, 57.25)	59.0 (44.0, 63.0)	.13
Females [%]	8 (40%)	4 (44%)	1.00
HbA1c [mmol/mol]	66.1 (11.4)	36.0 (2.1)	**<.01**
Weight [kg]	73.7 (15.7)	69.5 (11.4)	.48
Height [cm]	171.2 (9.4)	167.71 (12.5)	.42
PINP [ng/ml]	41.4 (34.6, 56.3)	69.4 (53.1, 76.6)	**<.01**
CTX-I [ng/ml]	0.24 (0.18, 0.44)	0.55 (0.49, 1.03)	**<.01**
TCNS	9.5 (7.0)	n.a.	**n.a.**

Significant P-values are displayed in bold font. T1DM, type 1 diabetes; CTX-I, C-terminal telopeptide of type I collagen; PINP, procollagen type 1 N-terminal propeptide; TCNS, Toronto Clinical Neuropathy Score; n.a., not applicable.

### Individuals with T1DM exhibited impaired bone remodeling as evidenced by lower Tb.F fractions

**Figure 1:**
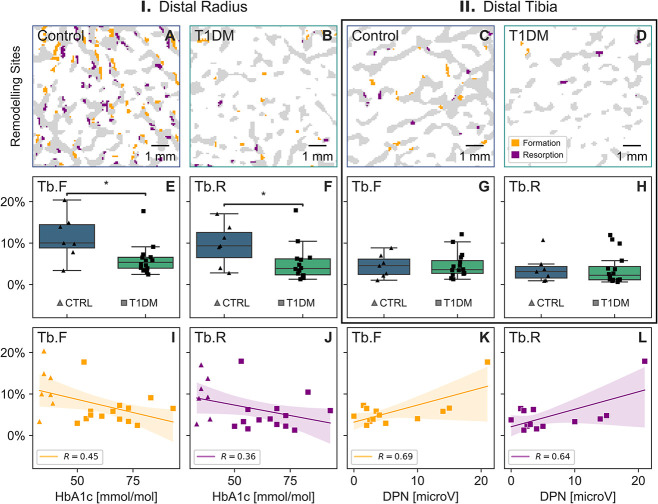
Bone remodeling sites quantified by trabecular bone formation (Tb.F) and resorption (Tb.R). Representative images show bone formation and resorption sites in trabecular bone microarchitecture assessed by HR-pQCT in controls (CTRL; A) and participants with type 1 diabetes (T1DM; B) at the distal radius, and in CTRL (C), and T1DM (D) at the distal tibia. Bone formation and resorption were measured at the distal radius (E, F, respectively) and at the distal tibia (G, H, respectively). Bone formation and resorption correlated with glycated hemoglobin (HbA1C; I, J, respectively) at the distal radius across all participants, and with DPN amplitude (K, L, respectively) in those with T1DM. Significant differences are indicated (^*^P < .05).

We used longitudinal HR-pQCT imaging to assess local bone remodeling at the distal radius and tibia (Table 2). Visual qualitative assessment showed distinct differences in bone remodeling between T1DM and CTRL (Figure 1A and B) and a relatively lower level of remodeling at the distal tibia in comparison to the radius (Figure 1C and D). We found that Tb.F was 47% lower and Tb.R 59% lower in T1DM compared with CTRL at the distal radius (*P* < .05, Figure 1E and F). No significant variations between groups (*P* > .5, Table 2) were observed at the distal tibia (Figure 1G and H). Bone remodeling at the distal radius correlated negatively with glycated hemoglobin (HbA1c) for Tb.F (*R* = −0.45, *P* < .05, Figure 1I) and Tb.R (*R* = −0.36, *P* = .11, Figure 1J) and positively with nerve conduction amplitude for Tb.F (*R* = 0.69, *P* < .01, Figure 1K) and Tb.R (*R* = 0.64, *P* < .05, Figure 1L) in participants with T1DM. We used biochemical bone turnover markers N-terminal PINP and CTX-I to assess bone formation and resorption, respectively. We found significantly lower levels of PINP and CTX-I in T1DM compared with CTRL (*P* < .01). However, we identified only a weak correlation between Tb.F and PINP (*R* = 0.35, *P* = .12), as well as between Tb.R and CTX-I (*R* = 0.25, *P* = .27), likely because of the limited sample size and the inherent noise levels in both parameters. Nevertheless, our results showed lower PINP and CTX in participants with T1DM compared with CTRL and corroborated the HR-pQCT findings of low bone remodeling in T1DM, particularly with increasing DSPN.

**Table 2:** HR-pQCT–based bone turnover and mechanoregulation markers as mean (standard deviation) for normally distributed variables and median (interquartile range) for non–normally distributed variables.

		**T1DM (*n* = 14)**	**CTRL (*n* = 7)**	***P*-value**
Distal radius	**Bone remodeling fractions**			
	Tb.F [%]	5.37 (3.97, 6.57)	10.04 (8.84, 14.43)	**.02**
	Tb.R [%]	3.87 (2.36, 6.19)	9.34 (6.51, 12.56)	**.04**
	Tb.F–Tb.R [%]	0.87 (1.11)	1.86 (1.63)	.12
	**Mechanoregulation**			
	OR_F_ [unitless]	1.15 (0.30)	1.75 (0.45)	**<.01**
	OR_R_ [unitless]	1.49 (1.35, 1.69)	1.81 (1.74, 2.17)	**.03**
	CCR [unitless]	0.39 (0.01)	0.4 (0.02)	.15
		**T1DM (*n* = 17)**	**CTRL (*n* = 7)**	***P*-value**
Distal tibia	**Bone remodeling fractions**			
	Tb.F [%]	3.58 (2.72, 5.76)	4.56 (2.45, 6.14)	.95
	Tb.R [%]	2.25 (1.16, 4.35)	3.15 (1.59, 4.35)	.79
	Tb.F–Tb.R [%]	1.31 (0.66, 1.63)	0.83 (0.37, 1.59)	.46
	**Mechanoregulation**			
	OR_F_ [unitless]	1.58 (0.55)	1.44 (0.39)	.55
	OR_R_ [unitless]	1.88 (1.63, 2.26)	1.48 (1.41, 1.75)	.11
	CCR [unitless]	0.40 (0.40, 0.41)	0.39 (0.39, 0.40)	.18

Significant P-values are displayed in bold font. T1DM, type 1 diabetes mellitus; Tb.F, trabecular bone formation; Tb.R, trabecular bone resorption; OR_F_, odds ratio formation; OR_R_, odds ratio resorption; CCR, correct classification rate.

Overall, the balance between bone formation and resorption (Tb.F–Tb.R) did not show significant differences across groups (*P* = .12). This suggests that although there were notable distinctions in bone remodeling fractions between the groups, there were no significant variations in net bone mineral density change over the study period. Furthermore, we did not observe any significant differences at the distal tibia, suggesting that regular loading at the distal tibia may be osteoprotective.

### In participants with T1DM, diminished responsiveness of bone formation to mechanical stimuli was observed

**Figure 2:**
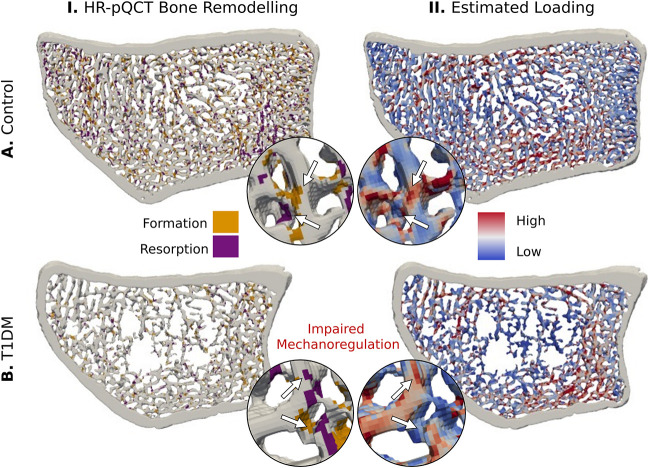
Visual representation of mechanoregulation over 4 yr in axial cross-sections (10 slices thick) of the human distal radius. (I) Sites of bone formation and resorption were determined using 3D image registration of baseline and 4–yr follow–up measurements for CTRL (A), and participants with T1DM (B). (II) corresponding estimated local mechanical loading visually shows higher strain energy density (SED) in regions of formation and lower SED in regions of resorbed bone.

**Figure 3:**
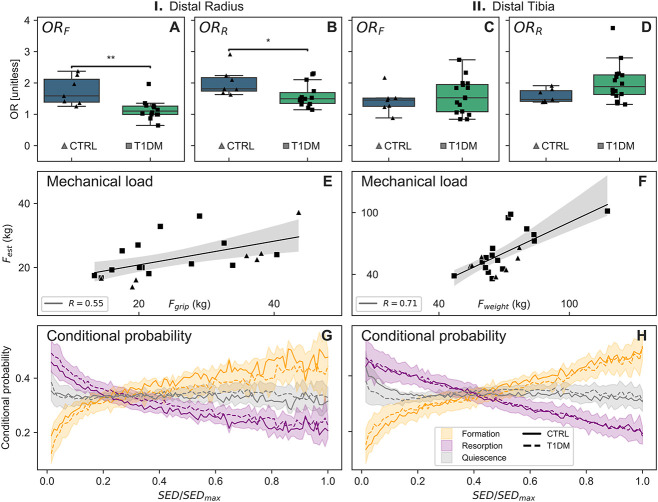
Quantitative assessment of bone mechanoregulation using longitudinal HR-pQCT *in vivo*. Within–participant odds ratios at the radius for bone formation (OR_F_; A) and resorption (OR_R_; B) and at the distal tibia for bone formation (OR_F_; C) and resorption (OR_R_; D). Mechanical loading was estimated using a computational load estimation algorithm that correlated with participant grip strength at the distal radius (E) and body weight at the distal tibia (F). Conditional probability of individual bone formation and resorption events was calculated at different strain levels and is shown for the distal radius (G) and distal tibia (H). Significant differences are indicated (^*^P < .05, ^*^^*^P < .01).

Using logarithmic regression between local remodeling events and the local *in vivo* mechanical signals (Figure 2), we calculated participant–specific odds ratios for formation (OR_F_) and resorption events (OR_R_) to occur with increasing strain. At the distal radius, we found individuals with T1DM had 34% lower OR_F_ (Table 2, Figure 3A, *P* < .01) and 18% lower OR_R_ (Table 2, Figure 3B, *P* < .05) compared with CTRL, indicating an impaired response to mechanical stimuli. No significant variations across groups (*P* > .05) were observed at the distal tibia (Figure 3C and D). Using the previously established computational method to estimate physiological loading from micro-FE, we assessed the loading conditions at the distal tibia and distal radius. Our analysis revealed no significant differences in estimated loading across groups (radius: *P* = .70, tibia: *P* = .15) but a positive correlation between the estimated loading at the radius and grip strength (Figure 3E, *R* = 0.55, *P* < .01), whereas the estimated loading at the tibia demonstrated a correlation with body weight (Figure 3F, *R* = 0.71, *P* < .01). These findings reaffirm that our algorithm estimates local mechanical stimuli that is reflective of expected day–to–day mechanical loading. We calculated conditional probability, associating the probability of remodeling events to occur at various strain levels, and found that bone was predominantly formed in high–strain and resorbed in low–strain regions (Figure 3G and H). From these probability curves, we derived a three–way CCR associating bone formation, quiescence, and resorption with high, medium, and low strain levels. Our analysis revealed that strain–driven remodeling events accounted for 39 ± 1% of bone remodeling events at the distal radius and 40 ± 1% at the distal tibia across all groups (Table 2).

## Discussion

This longitudinal cohort study advances our understanding of the mechanisms underlying skeletal dysfunction and compromised bone mechanoregulation in participants with T1DM and DSPN. We utilized longitudinal HR-pQCT imaging to assess dynamic bone remodeling and mechanoregulation markers in participants with T1DM and varying severity of diabetic DSPN, providing initial evidence *in vivo* that participants with T1DM not only show lower bone remodeling fractions but also impaired adaptation to mechanical loading. This may lead to weak spots in the bone microarchitecture and lead to bone fragility. Regular monitoring of bone health using HR-pQCT may be useful to detect and manage early changes in bone microarchitecture, particularly in participants who present with diabetic DSPN.

In our previous study, we reported findings of low bone remodeling fractions and preserved trabeculae in individuals with T1DM based on a cross–sectional analysis of this cohort.^9^ Building upon these initial observations, we have now expanded our investigation to include longitudinal data, allowing us to gain further insights into the dynamic skeletal effects of T1DM on trabecular bone. The role of mechanoregulation in bone fragility in T1DM has previously been suggested.^6^^,^^7^ Here, we present novel *in vivo* clinical evidence supporting this hypothesis. Recent *ex vivo* studies propose that this mechanoregulation impairment is because of an increased presence of mineralized lacunae and disruption of the osteocyte network caused by hyper–mineralized calcified matter in T1DM.^7^ While further longitudinal tracking of bone remodeling fractions and mechanoregulation is required to fully comprehend the relationship between bone remodeling and mechanical signals, our results confirm these *ex vivo* findings and suggest that pathologic osteocyte mechanoregulation may contribute to the low bone turnover observed in participants with T1DM. This is a critical insight since such impairment may prevent bone tissue from responding appropriately to mechanical strain, leading to microcrack formation.^7^ Coupled with low bone turnover, microcracks can accumulate and cause bone fragility, increasing the risk of fracture. Thus, independent from changes in trabecular bone structure, disturbed mechanoregulation may cause reduced bone adaptation and remodeling, leading to the accumulation of microcracks and bone fragility in T1DM participants.

Disrupted osteocyte mechanoregulation has previously been implicated in the low bone turnover observed in participants with T1DM. Prior research and meta-analyses have reported lower levels of markers for both bone formation and resorption in participants with T1DM, indicating alterations in the levels of sclerostin and osteoprotegerin may be responsible for this phenomenon.^29^ Specifically, sclerostin, which is produced by osteocytes, inhibits bone formation and indirectly reduces osteoclast activity by suppressing the secretion of osteoprotegerin.^30^ The diminished bone formation and resorption observed in participants with T1DM in this study are consistent with these previous studies. Furthermore, our results are in line with prior research examining biochemical bone turnover markers, demonstrating that inadequate glycaemic control was linked to diminished bone formation and resorption.^31–33^ We also found positive correlations between bone remodeling fractions at the distal radius and nerve conduction velocity, suggesting a positive association of nerve function on bone remodeling fractions. This aligns with the previous cross–sectional study, which demonstrated a positive correlation between cortical porosity and the severity of neuropathy.^9^ It is noteworthy that the impairment of bone mechanoregulation was not correlated with the degree of DSPN. This could suggest that diminished mechanoregulation is a consequence of T1DM, whereas DSPN may specifically impact bone remodeling fractions. However, the implications of these findings should be interpreted with caution because of the limited sample size of this study. Interventions targeting osteocyte mechanotransduction pathways and bone turnover, such as exercise and pharmacological therapies for sclerostin and osteoprotegerin, may help reduce the risk of fractures and prevent bone loss. Notably, monoclonal antibodies like romosozumab have demonstrated increased bone formation and reduced fracture risks in postmenopausal osteoporosis^34^ and may potentially improve bone remodeling. However, given their possible cardiovascular involvement further research is necessary to investigate their safety and efficacy in T1DM, especially since cardiovascular complications are common comorbidities in diabetes.

We observed that the negative effects of T1DM on bone remodeling fractions and mechanoregulation were more pronounced at the radius than the tibia, indicating that regular mechanical stimuli may counteract the adverse effects of T1DM on bone cells. In the cross–sectional analysis, we have reported an increase in cortical porosity at the tibia in participants with T1DM and no difference in the trabecular compartment at the radius.^9^ In this longitudinal analysis, more detailed evaluation enabled us to characterize the deficit in mechanoregulation in the trabecular compartment. The findings of the current study agree with our prior meta-analysis revealing that negative characteristics of bone microarchitecture were more severe at the radius than the tibia.^12^ Thus, mechanical loading may play an important role for bone health in individuals with T1DM, specifically at skeletal sites that are not naturally loaded day-to-day. While the effects of exercise on bone mechanoregulation are not well established, some studies have suggested that exercise can increase bone health in individuals with T1DM. A case–control study by Taylor et al.^35^ reported similar time–course changes in markers of bone formation but an attenuated suppression in bone resorption following moderate–intensity walking in adults with T1DM. Research conducted on healthy individuals has indicated that exercise regimens that maximize mechanical strain on the bone may be more effective in improving bone health,^36^^,^^37^ and starting exercise at a young age is critical to long–term bone health.^38^ Furthermore, the randomized controlled trial by Maggio et al.^38^ found that regular weight–bearing physical activity improved bone mineral deposition in children with T1DM to a similar extent as observed in skeletally healthy children. Therefore, this method of bone mechanoregulation represents a crucial step and has the potential to direct future research in determining the optimal type, duration, and intensity of exercise to maximize bone turnover in individuals with T1DM.

Our study has some limitations to consider when interpreting the implications of the results. The relatively small cohort limits definitive conclusions and increases the risk of type II errors because of limited power. However, our novel methodology provides exploratory evidence of potential relationships. Despite power constraints, the significant between–group differences after correction may reflect true effects worth larger validation. Importantly, the matched radius and tibia sites within each participant reduce variability when comparing these different skeletal locations. Although we cannot draw clinical implications from this sample size yet these preliminary data support previous cellular findings of impaired mechanoregulation in diabetes, especially with neuropathy. It should be noted that the participants with T1DM from the original cross–sectional cohort (*n* = 20) were pooled into a combined T1DM group for analysis because of a high rate of loss to follow-up during COVID-19. Specifically, groups with (*n* = 14) and without (*n* = 6) neuropathy could not be analyzed separately because of limited statistical power. Further research is essential to elucidate mechanisms and explore clinical translations suggested by these early results, as well as to separate the effects of diabetic neuropathy and diabetes.

Patient motion remains a significant challenge for longitudinal HR-pQCT investigations,^13^^,^^14^^,^^39^ as we had to exclude several participants because of this issue. Future advances in motion suppression, whether through computational or hardware approaches, will be necessary to implement HR-pQCT–based bone remodeling methods in clinical screening.^24^ Furthermore, our study had some computational limitations. The linear transformation used after image registration may have introduced interpolation artifacts. We conducted preliminary tests to explore the potential benefits of higher–order interpolations; however, the results showed only minor improvements in outcomes at the expense of significantly increased computational time.^15^ Additionally, the load estimation model did not account for nonlinear behavior, viscoelastic effects, or disease–specific material models for diabetes. Nonetheless, only minor, linear–elastic deformations occur during daily activities, and therefore, we do not expect these limitations to significantly affect our findings.^16^ Furthermore, the mechanoregulation analysis examined relative differences in strain patterns, which should be independent of absolute strain magnitudes or tissue properties. Overall, while our study had its limitations, it represents a significant step forward in unraveling the underlying causes of bone fragility in T1DM and highlights the need for larger–scale studies with more diverse populations.

This initial investigation of skeletal dysfunction in T1DM has provided early mechanistic insights into potential impairments in bone mechanoregulation and turnover. While limited by sample size, the findings suggest decreased remodeling rates, and impaired spatial correlations between tissue–level strains and bone remodeling sites may contribute to bone fragility in T1DM, particularly with diabetic DSPN. These preliminary observations further our fundamental understanding of how bone mechanoregulation may be disrupted in diabetes. Additional research in larger cohorts is necessary to fully understand these mechanisms and explore how assessing bone quality and mechanoregulation could inform exercise or pharmacological interventions targeting bone health.

## Supplementary Material

230712_JBMR_Supplement_zjad014

## Data Availability

The data and analytic code for this study may be made available from the corresponding author upon reasonable request.
