# Supplementary material for: Bone remodeling and responsiveness to mechanical stimuli in individuals with type 1 diabetes mellitus
Source: J Bone Miner Res. 2024 Jan 4;39(2):85–94. doi: 10.1093/jbmr/zjad014 (PMC11340785; doi:10.1093/jbmr/zjad014)
Supplement: 230712_JBMR_Supplement_zjad014 [file 230712_JBMR_Supplement_zjad014.docx]

**Table S1.**Study population characteristics at the **distal radius**, biochemical bone turnover markers, and neuropathy assessment as mean (standard deviation) at baseline for normally distributed variables and median (interquartile range) for non-normally distributed variables.

|  | T1DM (n=14) | CTRL (n=7) | p-value |  |
| --- | --- | --- | --- | --- |
| Age [years] | 50.5 (41.5, 56.5) | 59.0 (47.0, 62.5) | 0.35 |  |
| Females [%] | 5 (36%) | 3 (43%) | 1 |  |
| HbA1c [mmol/mol] | 66.64 (12.54) | 36.0 (2.31) | **<0.01** |  |
| Weight [kg] | 77.63 (16.56) | 70.76 (12.41) | 0.35 |  |
| Height [cm] | 171.73 (9.9) | 170.43 (12.92) | 0.8 |  |
| P1NP [ng/ml] | 40.72 (33.94, 52.63) | 71.63 (59.5, 84.83) | **<0.01** |  |
| CTX-I [ng/ml] | 0.22 (0.15, 0.49) | 0.86 (0.52, 1.04) | **<0.01** |  |
| TCNS | 10.14 (7.72) | n.a. | **n.a.** |  |
|  | T1DM = type 1 diabetes mellitus, CTX-I = C-terminal telopeptide of type I collagen, P1NP = procollagen type 1 N-terminal propeptide, TCNS = Toronto Clinical Neuropathy Score, n.a. = not applicable. | | | |

**Table S2.**Study population characteristics at the **distal tibia**, biochemical bone turnover markers, and neuropathy assessment as mean (standard deviation) at baseline for normally distributed variables and median (interquartile range) for non-normally distributed variables.

|  | T1DM (n=17) | CTRL (n=7) | p-value |  |
| --- | --- | --- | --- | --- |
| Age [years] | 48.0 (33.0, 58.0) | 62.0 (58.5, 63.5) | **0.04** |  |
| Females [%] | 7 (41%) | 4 (57%) | 0.79 |  |
| HbA1c [mmol/mol] | 64.47 (8.42) | 36.43 (2.15) | **<0.01** |  |
| Weight [kg] | 67.0 (62.7, 80.4) | 66.2 (57.4, 73.6) | 0.36 |  |
| Height [cm] | 170.67 (9.04) | 163.33 (10.35) | 0.1 |  |
| P1NP [ng/ml] | 42.54 (34.89, 55.48) | 69.42 (60.38, 82.34) | **<0.01** |  |
| CTX-I [ng/ml] | 0.31 (0.2) | 0.72 (0.27) | **<0.01** |  |
| TCNS | 8.18 (6.17) | n.a. | **n.a.** |  |
|  | T1DM = type 1 diabetes mellitus, CTX-I = C-terminal telopeptide of type I collagen, P1NP = procollagen type 1 N-terminal propeptide, TCNS = Toronto Clinical Neuropathy Score, n.a. = not applicable. | | | |
